# Assessing lung cancer progression and survival with infrared spectroscopy of blood serum

**DOI:** 10.1186/s12916-025-03924-3

**Published:** 2025-02-21

**Authors:** Kosmas V. Kepesidis, Mircea-Gabriel Stoleriu, Nico Feiler, Lea Gigou, Frank Fleischmann, Jacqueline Aschauer, Sabine Eiselen, Ina Koch, Niels Reinmuth, Amanda Tufman, Jürgen Behr, Mihaela Žigman

**Affiliations:** 1 Chair of Experimental Physics - Laser Physics, Ludwig-Maximilians-Universität München (LMU), Garching, Germany; 2 Laboratory for Attosecond Physics, Max Planck Institute of Quantum Optics (MPQ), Garching, Germany; 3 Center for Molecular Fingerprinting (CMF), Budapest, Hungary; 4 Asklepios Biobank for Lung Diseases, Department of Thoracic Surgery, Member of the German Center for Lung Research, DZL, Asklepios Fachkliniken München-Gauting, Munich, Germany; 5 Department of Medicine V, LMU University Hospital, LMU Munich, Member of the German Center for Lung Research, Munich, Germany

**Keywords:** Lung cancer, Infrared molecular fingerprinting, Survival analysis, Prognostic biomarker, Liquid biopsy

## Abstract

**Background:**

Infrared molecular fingerprinting has been identified as a new minimally invasive technological tool for disease diagnosis. While the utility of cross-molecular infrared fingerprints of serum and plasma for in vitro cancer diagnostics has been recently demonstrated, their potential for stratifying and predicting the prognosis of lung cancer remained unexplored. This study investigates the capability of this approach to predict survival and stratify lung cancer patients.

**Methods:**

Molecular fingerprinting through vibrational spectroscopy is employed to probe lung cancer. Fourier-transform infrared (FTIR) spectroscopy is applied to blood sera from 160 therapy-naive lung cancer patients, who were followed for up to 4 years. Machine learning is then utilized to evaluate the prognostic utility of this new approach. Additionally, a case-control study involving 501 individuals is analyzed to investigate the relationship between FTIR spectra and disease progression.

**Results:**

Overall, we establish a strong correlation between the infrared fingerprints and disease progression, specifically in terms of tumor stage. Furthermore, we demonstrate that infrared fingerprinting provides insights into patient survival at performance levels comparable to those of tumor stage and relevant blood-based biomarkers.

**Conclusions:**

Identifying the combined capacity of infrared fingerprinting to complement primary lung cancer diagnostics and to assist in the assessment of lung cancer survival represents the first proof-of-concept study underscoring the potential of this profiling platform. This may provide new avenues for the development of tailored, personalized treatment decision-making.

**Supplementary Information:**

The online version contains supplementary material available at 10.1186/s12916-025-03924-3.

## Background

Lung cancer remains a major cause of cancer-related mortality worldwide [1], necessitating the development of robust diagnostic and prognostic tools to guide treatment decisions and improve patient outcomes. Its high mortality is frequently associated with aggressive histological subtypes and late diagnosis with consecutively limited treatment options. Thus, efforts to improve prevention, early detection, and treatment outcomes are crucial to reducing the burden of this disease.

A variety of promising lung cancer molecular candidate biomarkers (autoantibodies, complement fragments, microRNAs, circulating tumor DNA, DNA methylation, blood protein and metabolite profiling) [2–7] have been demonstrated to aid diagnosis of lung cancer, either in combination or in the absence of low-dose computed tomography (LDCT) screening [8]. However, the diagnostic value, clinical cost-effectiveness, analysis time and need for repetitive procedures remain relevant factors limiting the implementation of available approaches for clinical routine [9].

For these reasons, there is an unmet need for additional less-invasive diagnostic tests to facilitate prognostic stratification in patients with lung cancer [10]. Given the advantages of infrared molecular spectroscopy for in vitro analytical profiling [11], the approach has the potential in the detection of cancer [12]. Infrared (IR) spectroscopy detects how molecules absorb IR radiation at frequencies matching their vibration modes. These unique absorption patterns, characteristic of molecular structures, are recorded in the IR spectrum, providing a molecular overview of the sample. The method is fast, cost-effective, and label-free. The advantages of label-free fingerprinting include reduced sample preparation time and costs, minimizing the potential for artifacts introduced by labeling agents, preserving the native state of the sample, simplifying experimental implementation, and enabling real-time profiling. When applied to blood serum or plasma samples, it delivers an infrared molecular fingerprint (IMF) reflecting the chemical composition of a sample, that is, the person’s molecular blood phenotype [13, 14]. It provides a discovery platform with opportunities to identify further biomarkers for lung cancer detection [15].

This blood-based, label-free, cost-feasible, and time-efficient approach to delivering IMFs could be utilized to build classification models for the stratification of lung cancer. It was this far only used for the detection of many solid tumors in pilot studies involving brain [16–18], breast [12, 19–23], bladder [12, 24], lung [12, 25], prostate [12, 26], and other cancer entities [24, 27, 28], with some of the studies reporting very high sensitivities and specificity values [16, 19, 21, 24, 26, 27].

Our previous studies have established molecular profiling of venous blood serum and plasma with infrared fingerprinting, aided by machine learning, as a new tool to detect lung cancer [12]. We also demonstrated high robustness and the ease of infrared bulk fluid measurements, along with its low costs and time efficiency [13], and the possibility to expand the understanding of infrared fingerprints to individual molecules [15].

Relevant to lung cancer in vitro diagnostics, our previous study revealed that IMFs correlate with tumor size [12]. However, due to the smaller cohort size available, we were previously not in a position to assess the possible correlation between the blood-based IMFs and relevant tumor, node, metastasis (TNM) staging that is essential for initial diagnosis and treatment planning of lung cancer.

Building on the established, here we evaluate the value of infrared fingerprinting as a possible new platform technology to asses TNM stage and patient’s survival rates at therapy-naive states, along with established blood-based markers. To the best of our knowledge, our study evaluated for the first time the relationship between IMFs and lung cancer patient survival that could be beneficial to further improve clinical diagnosis, treatment, and survival of lung cancer patients [29].

The current study aimed to analyze the relationship between IMFs, lung cancer stages, and patient survival along with further clinical parameters. For this reason, we: i) used a well-characterized large-volume cohort of lung cancer patients undergoing curative resections or palliative chemotherapy, ii) generated IMFs of blood sera reflecting the underlying multi-molecular physiological mechanisms of the disease, iii) investigated the correlation between IMF and disease progression, and iv) assessed the information content of IMFs concerning overall survival by comprehensive statistical survival modeling.

The capacity of IMF to predict lung cancer survival, as well as its relation to disease progression, may provide additional information for personalized diagnosis and treatment.

## Methods

### Ethics statement

This study is based on blood samples of lung cancer patients derived from the Asklepios biobank of lung diseases under project number 333-10 and study protocol number 17–141. Included control individuals provided written informed consent for the study under research study protocol number 17–182. Both research protocols were approved by the Ethics Committee of the Ludwig-Maximillian-University (LMU) of Munich. Our study complies with all relevant ethical regulations and was conducted according to Good Clinical Practice (ICH-GCP) and the principles of the Declaration of Helsinki. The clinical trial is registered (ID DRKS00013217) at the German Clinical Trials Register (DRKS). The following clinical centers were involved in subject recruitment and sample collections of the clinical study: the Department of Internal Medicine V for Pneumology and the Asklepios Lung Clinic (Gauting), affiliated with the Comprehensive Pneumology Centre (CPC) Munich, and the German Centre for Lung Research, DZL; the Department of Urology and the Department of Obstetrics and Gynecology, LMU University Hospital, LMU Munich. Information on the full breakdown of study participants presented is listed in Table 1.. From the existing dataset, the recorded IMFs were selected for further analysis according to the following criteria: Only data from cancer patients with clinically confirmed carcinoma of the lung, before any cancer-related therapy were considered.

**Table 1:** Population characteristics of the cohort and case-control studies

Censored	# patients	Age (years)	% Female	BMI (kg/m²)		**Stage I**				
Yes	84	68 ± 9	46	25 ± 4		Group	# individuals	Age (years)	% Female	BMI (kg/m²)
No	76	70 ± 11	42	26 ± 5		Cases	39	70 ± 9	44	26 ± 6
						Controls	39	70 ± 9	46	27 ± 5
Censored	Operable tumor	Non-operable tumor	N/A							
Yes	59	25	0			**Stage II**				
No	20	54	2			Group	# individuals	Age (years)	% Female	BMI (kg/m²)
						Cases	33	69 ± 8	36	25 ± 4
Censored	Stage I	Stage II	Stage III	Stage IV	N/A	Controls	33	69 ± 8	36	28 ± 5
Yes	11	7	13	9	44					
No	6	2	19	28	21	**Stage III**				
						Group	# individuals	Age (years)	% Female	BMI (kg/m²)
Censored	AC	SCC	LCC	N/A		Cases	94	68 ± 10	44	26 ± 5
Yes	58	24	1	1		Controls	94	68 ± 10	43	27 ± 4
No	46	20	6	4						
						**Stage IV**				
						Group	# individuals	Age (years)	% Female	BMI (kg/m²)
						Cases	151	68 ± 9	52	25 ± 5
						controls	151	68 ± 9	51	26 ± 4

First sub-table: Characteristics of the lung cancer cohort used in the survival analysis. The first part shows the breakdown of lung cancer patients in terms of demographics. The second part presents information on performed surgery during the study. The third part shows tumor staging information according to the TNM Classification of Malignant Tumors (Union for International Cancer Control (UICC)). The last part shows the breakdown of patients in terms of tumor histology. In Additional file 1: Tables S1-S3, we provide additional information on the cohort breakdown

Second sub-table: Characteristics of the case-control study used in the classification analysis for disease detection. Cancer cases were stratified in terms of tumor stage and matched to appropriate controls in terms of age and sex

### Study cohort characteristics

For the survival analysis, in total 160 (predominantly) non-small-cell lung cancer (NSCLC) patients were enrolled in this study (see left part of Table 1.). Out of them, 84 were censored. The two groups exhibit similarities in terms of their age, gender, and body-mass-index (BMI) distributions, as shown in Table 1. (first part of left subtable). Information on performed surgery during the study was collected in follow-up visits. This information is presented in 1 (second part of left sub-table). Full staging information according to the TNM Classification of Malignant Tumors (Union for International Cancer Control (UICC) [30], was captured only partially. Information on the distribution of stages for both the censored and not censored patient groups is presented in Table 1. (third part of left sub-table). Patients with unavailable staging information were excluded from the stage-related analysis but were included in all other evaluations. In terms of tumor histology [30], a relative balance is observed between squamous cell carcinoma (SCC) and adenocarcinoma (AC). Also, a few cases of large-cell carcinoma (LCC) were enrolled. While the cohort consists predominantly of these three histological subtypes of NSCLC, patients with rare or unknown histological subtypes are present as well (see last part of Table 1.).

For the classification analysis in terms of stage, a total number of 501 individuals were included in the case-control study. This corresponds to the 317 cases and 184 healthy control individuals. Information on study participants is presented in Table 1. (right sub-table). It needs to be stated that 133 healthy individuals were included as controls in more than one case-control design for different stages. For the classification analysis, the lung cancer cases were stratified in terms of tumor stage and matched to appropriate control individuals based on sex, age, and BMI.

### Study setup and workflow

**Fig. 1:**
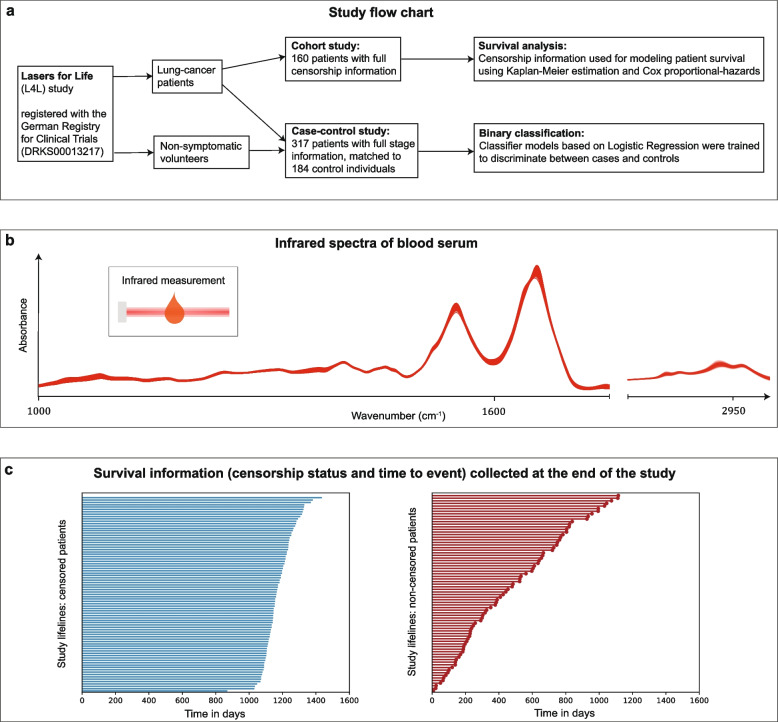
Outline of study design and workflow and main results. **a** Flow chart showing the structure of the study. **b** Infrared spectra of blood sera. The plot shows absorbance per wave number, in the mid-infrared domain, for all lung cancer patients involved in the study. **c** Study lifelines of participating patients, indicating i) the time from the blood sampling at the primary lung cancer diagnosis until the end of the study for censored patients and ii) until the time of death for non-censored study participants

This study aims to assess the information content of blood-based IMF for disease progression and survival outcomes among lung cancer patients. For this reason, two studies (cohort and case-control) were designed (see Fig. 1a).

A systematic approach was used to select eligible patients and also include matched controls for the study. Individuals who were diagnosed with lung cancer were enrolled, and samples were collected before any cancer-related treatment. Relevant clinical and pathological data from medical records were collected, including patient demographics, tumor characteristics, treatment regimens, and follow-up information. Blood sera were collected according to well-defined standard operating procedures to minimize pre-analytical errors [13]. An automated sample delivery system was applied for high-throughput, high-reproducibility, and cost-efficient infrared fingerprinting of liquid sera with an FTIR spectrometer (see Fig. 1b).

Inclusion criteria encompassed patients diagnosed with primary lung cancer, before any cancer-related treatment and regardless of the cancer stage. For the survival analysis, patients who reached the end of the study without experiencing their death were classified as censored [31]. On the other hand, patients whose death was observed during the study were classified as non-censored. Patients with missing or incomplete censorship status were excluded from the study. A total of 160 lung cancer patients were included in this cohort study. A case-control study was constructed to investigate disease progression. Patients with missing or incomplete staging information were excluded to ensure the integrity of the analysis. 317 lung cancer patients met the inclusion criteria and were included in the final analysis.

Comprehensive data on patient demographics, clinical characteristics, tumor features, and treatment details were collected and managed in a standardized fashion. This involved reviewing medical records, pathology reports, and treatment data. Data variables of interest included age, gender, BMI, tumor stage, histological subtype, cancer-related treatment modalities (e.g., surgery, chemotherapy), and survival outcomes (overall survival). Patient follow-ups were conducted on-site during post-treatments according to the standard clinical protocol. Survival outcomes (overall survival) were recorded at the end of the study, approximately 4 years after the start (see Fig. 1c). Overall patient survival was defined as the time from diagnosis until death from any cause or the last follow-up visit. Patients who were either alive at the end of the study (and at the start of data analysis) or lost to follow-up were classified as censored. Non-censored patients were individuals for whom we could identify the date of death and thus evaluate the duration of survival from the time of sampling and primary diagnosis.

Statistical analysis was performed using appropriate survival analysis methods to evaluate the relationship between various factors (cancer stage, biomarkers, IMF, etc.) and patient survival outcomes. Kaplan-Meier survival curves were generated to estimate survival probabilities over time, stratified by relevant variables such as tumor stage, treatment modality, and tumor histology. Log-rank tests were conducted to compare survival curves and assess the significance of differences between subgroups. To identify independent prognostic factors associated with survival outcomes, multivariable Cox proportional hazards regression analysis was performed. Apart from IMF, this analysis also considered various covariates, including age, gender, tumor stage, and molecular markers captured via clinical chemistry. Corresponding 95% confidence intervals (CIs) were calculated to quantify the strength of associations between each covariate and survival outcomes.

In addition to survival analysis, a larger case-control study (similar to our previous work [12]) was designed to evaluate the performance of IMF-based classification models concerning disease diagnosis and investigate the effect of disease progression on the corresponding ROC curves. In this study, healthy (asymptomatic) volunteers were included as controls. Control individuals were statistically matched to the lung cancer cases based on age, gender, and BMI.

### Sample collection and storage

Venous blood samples were collected, processed to sera, and stored according to the same standard operating procedures. Blood draws were all performed using Safety-Multifly needles of at least 21 G (Sarstedt) and collected with 4.9 ml serum Monovettes (Sarstedt). Before centrifugation, serum tubes were stored upright for at least 20 minutes to ensure blood coagulation. Centrifugation was performed at 2000 g for 10 min at 20°C within three hours after blood donation. The supernatant was manually aliquoted into 0.5 ml fractions and frozen within two hours after centrifugation. Samples were stored at −80°C in the clinics, transported to the analytical laboratory on dry ice, and again stored at −80°C until sample pre-processing.

### Sample handling and FTIR measurements

To prepare samples for FTIR measurements, one 0.5 ml aliquot of serum per sample was thawed in a water bath at 4°C, carefully vortexed, and again centrifuged for 10 min at 2000 g. Subsequently, four to six small-volume aliquots were generated and refrozen at −80°C. The volume of these aliquots varied over time between 50 $\upmu$L and 90 $\upmu$L. Independently of the aliquoted volume, 35 $\upmu$L of the sample was injected into the measurement cuvette. The 90 $\upmu$l aliquots allowed direct re-measurement in FTIR in case of any instrument issue. All the FTIR measurements were performed upon two freeze-thaw cycles. The samples were measured in a fully randomized order together with other samples. The samples were aliquoted and measured in a blinded fashion, that is, the person performing the measurements had no access to the clinical information of the study participants. For infrared spectroscopic measurements, a commercial FTIR device specialized in the analysis of liquid samples in transmission mode was used (MIRA-Analyzer, CLADE GmbH, formally known as Micro-Biolytics GmbH). The flow-through transmission cuvette was made of CaF2 windows with 8 $\upmu$m optical path length. The spectra were acquired with a resolution of 4 cm^−1^ in a spectral range between 950 cm^−1^ and 3050 cm^−1^. A water reference spectrum was recorded automatically after each sample measurement to reconstruct the IR absorption spectra. The actual path length was also determined automatically at each measurement, and the spectra were adjusted accordingly. FTIR measurements were performed in batches of 25 samples with a quality control serum (pooled human serum, BioWest, Nuaillé, France) measured at the beginning of the batch and after five samples, each, resulting in a batch size of 31 samples. The use of the quality control samples allowed tracking of potential technical errors and drifts over the entire measurement period [32]. If an air bubble was present during the measurement, this was immediately noticeable by the saturation of the detector. In such cases, the measurement was considered faulty. For 90$\upmu$L aliquots, the measurement was repeated at the end of the batch using the original aliquot. For 50$\upmu$L aliquots, a new aliquot had to be thawed. Those re-measurements were typically performed in one of the next batches.

### Pre-processing of infrared absorption spectra

The MIRA analyzer tends to overcompensate the sample’s infrared spectrum concerning water, as plasma contains about 10 percent of solid matter. For that reason, a preprocessing was performed according to our previous work [12]. In brief, a reference water spectrum was added to undo the overcompensation, spectra were truncated to 1000–3000 cm^−1^ and the ‘silent region’, between 1850 cm^−1^ and 2800 cm^−1^, was removed. Finally, all spectra were normalized using Euclidean (L2) norm.

### Survival analysis methods

The survival analysis was performed using the lifelines package (v.0.27.4) [33]. For estimating the survival probability function, the Kaplan-Meier method was used. Kaplan-Meier estimator is one of the most widely used non-parametric techniques for modeling survival distributions [34]. For testing the significance of differences between survival functions, the log-rank test was used. The log-rank test is a large sample chi-square ($\chi ^2$) test that compares two or more Kaplan-Meier curves. To build predictive models based on multiple spectral features, the Cox proportional hazards method was used [34]. The evaluations were performed within a 10-fold cross-validation using Harrell’s C-index (also known as the concordance index) as the performance metric. Within each split, a grid search was conducted on the training fold to explore various hyperparameter combinations. For each hyperparameter combination, we used a 3-fold cross-validation nested inside the grid search to evaluate Harrell’s C-index. Subsequently, the best-performing model on the training fold was assessed on the test fold using Harrell’s C-index once again. C-index is a goodness of fit measure for models which produce risk scores. It is commonly used to evaluate risk models in survival analysis, where data may be censored [35–37]. Values of C-index near 0.5 indicate that the risk score predictions are no better than a coin flip in determining which patient will live longer. Values near 1 indicate that the risk scores are good at determining which of two patients will have the disease first.

### Statistical matching in case-control study

Achieving covariate balance between cases and controls is crucial in observational studies to neutralize confounding factors and minimize bias. In this study, we matched cases and controls based on sex, age, and BMI using optimal pair matching with Mahalanobis distance within propensity score calipers [38]. The implementation was carried out in R (v. 3.5.1).

### Binary classification and ROC curves

To derive classification models, we used Scikit-Learn (v.1.1.3) [39], an open-source machine learning framework in Python (v.3.9.13). We trained binary classification models using logistic regression. We trained binary classification models using logistic regression. To properly validate our models, we did a performance evaluation with 10-fold cross-validation, visualizing the results with ROC curves. The models were regularized using L2 norm regularization, with the corresponding regularization factor determined through grid search within the cross-validation process. The results of the cross-validation are reported in terms of descriptive statistics, that is, the mean and the standard deviation of the resulting distribution of AUC values and mean ROC curves.

### Differential fingerprints

To visualize the information patterns associated with differences between cases and controls in a case-control setting, we employed the concept of differential fingerprints, as described in previous studies [12, 13, 15]. Differential fingerprints represent the mean difference per wavenumber between cases (e.g., lung cancer patients) and control individuals. The shaded area on the background corresponds to the standard deviation of the controls. A larger magnitude of the differential fingerprint indicates a greater difference between cases and controls, resulting in a stronger difference of spectroscopically measured information for a machine learning algorithm to use in distinguishing between the two groups.

### Binormal model for ROC curves

The influence of disease progression on the ROC curve was modeled in Stata (v. 17.0) using the package st0155 [40]. Assuming that the distributions of classification scores underlying the ROC curve are transformable to normality using a strictly increasing transformation, the classical model of binormal ROC curves can be applied: $\text {ROC}(t) = \Phi (\alpha _0 + \alpha _1 \Phi ^{-1}(t))$, where $\Phi$ is the standard normal cumulative distribution function, *t* is the false positive rate, and $\alpha _0$ and $\alpha _1$ are the intercept and slope of the curve, respectively. Using this framework, the ROC curve was modeled as a function of covariates using the approach of generalized linear models (GLM) for ROC curves, known as ROC-GLM regression [41]. Mathematically, models of this kind have the following form: $g(\text {ROC}_Z(t)) = h_0(t) + \beta Z$, where $h_0(\cdot )$ is the baseline function, $g(\cdot )$ is the link function, and *Z* is the covariate vector with corresponding parameters $\beta$. Both $h_0(\cdot )$ and $g(\cdot )$ are monotone increasing (or decreasing) functions on (0, 1) such as $\Phi$, ensuring the GLM represents a ROC curve. The GLM was fitted using a semiparametric approach and parameter estimates for the covariate’s influence on intercept and slope were evaluated by bootstrapping (1000 bootstrap samples, separate sampling from cases and controls). The significance of the estimates was assessed through *p*-values calculated from Wald statistics [40]. The classification scores underlying this analysis were obtained from a support vector classifier trained and evaluated using leave-one-out cross-validation in Scikit-Learn [39].

### Learning curves

To compute learning curves, the classifier under investigation was trained on randomly selected subsets of varying sizes of the available data. For each subset, the performance was evaluated using the AUC. This procedure was repeated 5 times for each sample size and the results averaged. Then, following [42], an inverse power law model was fitted to the obtained learning curves.

## Results

### Patient survival analysis

As a first step, we quantitatively investigate survival rates in our cohort, without the involvement of IMF. For this, an established Kaplan-Maier (KM) estimator, one of the most widely used non-parametric techniques for modeling survival distributions, was applied.

**Fig. 2:**
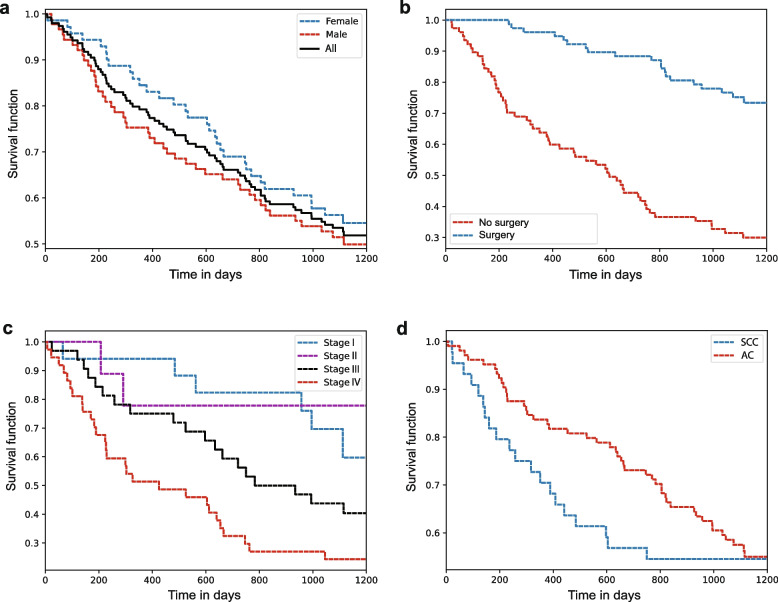
Survival analysis based on single covariate using Kaplan-Meier estimation. **a** Survival functions for all study patients, and survival functions separately by gender status (female - blue line; male - red line; combined - black line). **b** Survival functions for patients with operable (blue line) and non-operable (red line) lung cancer tumors. **c** Stratified survival functions by TNM staging (stage I - blue; stage II - violet; stage II - black; stage IV - red line). **d** Stratified survival functions concerning lung cancer tumor histology (squamous cell carcinoma - blue line; adenocarcinoma - red line)

Figure 2 shows the resulting survival probability as a function of time concerning different levels of nominal and ordinal covariates. To provide a general overview of the study, we apply the survival function for all study patients involved, and further provide survival in dependence of gender status (Fig. 2a). In this case, the two gender-specific curves deviate only slightly from the baseline (pulled analysis). A statistical comparison between the two gender-specific curves, based on log-rank testing (see Methods section), yielded a large *p*-value of $p=0.41$. Comparing the gender-specific survival curves of our cohort with the survival rates for the whole of Germany as provided by the Robert Koch Institute [43], these very results reflect comparable trends. Women still have slightly better overall survival than men, independent from the UICC stage at the time of primary diagnosis. However, it is known that the gender-specific survival rates progress in opposite directions: For a few decades now, the survival rates for females have a declining rate, whereas the rates for males have risen continuously over the same period and now have come close to those of females. This different development can be attributed to the change in smoking habits since the end of the 1990s and is likely to continue.

We further investigated whether the survival function of individuals reveals any difference for patients with operable versus non-operable tumors (see Fig. 2b). As expected, we find that the differences are significant. Performing statistical significance testing yields a *p*-value of $p < 0.005$. This means that patients with operable tumors have significantly larger survival chances. When investigating whether the TNM stage correlates with survival, we expectedly found that individuals with primary diagnosis at lower TNM stage survive longer than individuals where the TNM stage was higher at the time of primary diagnosis (see Fig. 2c). The significance comparison in this case revealed significant differences between stage IV and the rest of the stages. A detailed presentation of the related *p*-values is further given in Additional file 1: Table S4. This observation on the existing cohort reflects the trends in Germany, where the survival curves also decrease dramatically with advanced UICC stages: The 5-year relative survival for UICC stage I patients is 73 percent for females and 63 percent for males, whereas only 7 percent of females and 4 percent of males live longer than 5 years after being diagnosed with a metastasizing stage IV lung carcinoma [43].

### Infrared fingerprints and lung cancer progression

**Fig. 3:**
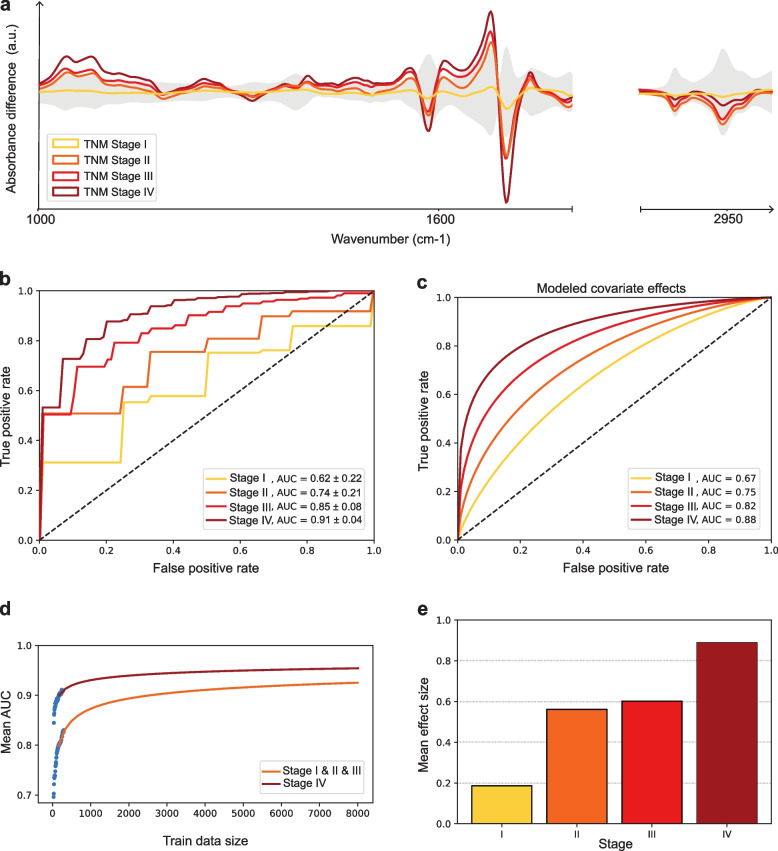
Assessment of information content of IMF concerning disease progression. **a** Differential infrared spectra, showing the mean difference per wavenumber between measured data of lung cancer patients of different stages and age- and gender-matched healthy control individuals. The shaded area corresponds to the standard deviation per wavenumber in the control group. **b** Empirical ROC curves (and the corresponding AUC values) for the binary classification between lung cancer patients (of different stages) and matched healthy control individuals. The classification was performed using logistic regression within 10-fold cross-validation. **c** Modeled covariate effect of the predicted lung tumor stage on the ROC curve using a generalized linear model (ROC-GLM). See the Methods section for more details. **d** Learning curves for mean AUC, for the binary classification between lung cancer patients and matched healthy control individuals (experimental data depicted as blue dots; Stage I, II, III - orange fit line; Stage IV - dark red fit line). **e** Mean effect size indicating the overall average differences between infrared fingerprints of cases (of different stages) and controls

We previously showed that the size of the main tumor diameter in lung cancer patients correlates with IMF deviations [12]. In the current study, we set out to specifically investigate whether the deviations of IMF correlate with the TNM stages at diagnosis in the population under investigation. Figure 3 shows the strong relation between the IMFs and lung cancer progression. Specifically, in Fig. 3a we plot the differential fingerprint for different matched lung cancer case-control groups (controls are non-symptomatic healthy volunteers), stratified in terms of the TNM stage. We observe that disease-related patterns span a broad spectral range. In particular, the range between 1000 cm^−1^ and 1700 cm^−1^ contains informative patterns related to numerous potential biomolecules. This region has been conventionally associated with the absorbance of proteins and carbohydrates, including glycosylated proteins. Thus, changes in this region could be due to alterations in their concentration, structure, or glycosylation pattern. Most importantly, our results reveal that the strength of the lung cancer pattern in the blood serum, as measured via blood serum IMFs, correlates with the tumor stage along the entire range of observed wavenumbers. This is evident in the differential fingerprints, where the amplitude of the observed pattern consistently increases with disease progression while the overall pattern remains unchanged. To further investigate this relationship, using the created stratified matched cohorts, we build classification models to evaluate the capacity to distinguish between lung cancer cases and control individuals separately for each tumor TNM stage. Figure 3b shows the resulting empirical ROC curves for each stage. The observed relation between the strength of the differential fingerprint and disease progression propagates to the predictive models. Although stage II lung cancer patients can be classified with an AUC of ROC higher than 0.70, we observe a further increase in the capacity of the candidate medical test to detect lung cancer with higher stages. However, from this analysis, it cannot be determined whether the increase in test performance is caused by disease progression or an increased number of available samples for stages III and IV. To further address and investigate this, we proceed with rigorous modeling of the effect of stage on the ROC curve. Specifically, we make use of the so-called binormal model of the ROC curve [41]. This model allows for rigorous investigation of covariate effects (in this case the TNM stage) via generalized linear regression models (GLM). The resulting binormal ROC curves displayed in Fig. 3c show a trend fully consistent with the empirical results. Furthermore, we calculated the *p*-value for the hypothesis that the coefficient $\beta$, which captures the influence of the lung cancer stage on the binormal ROC curve (see Methods section), is zero. Since this *p*-value is well below 0.05, we can reject the hypothesis and conclude that the influence of the lung cancer stage on the ROC curve is significant. Thus, we uncover a systematic increase of a possible IMF-based diagnostic test for lung cancer detection to detect lung tumors with higher efficiency when already progressed to later stages.

Our results provide a relevant extension as well as partially independent confirmation of the previously published study on correlating tumor size and the extent of infrared fingerprint aberrations [12] – that the IMF captures tumor-specific patterns in blood sera. As the size of the training data increases, machine-learning models tend to exhibit greater robustness. Consequently, one can anticipate an increase in the AUC for each specific disease stage. In Fig. 3d, we seek to illustrate this phenomenon by employing a learning curve fitted with a saturation function, as detailed in a previously published work [42]. We observe that, with the increased size of train data, the detection performance for non-metastatic as well as stage IV (metastatic) cases saturate above AUC=0.9. Nevertheless, the disparity in final AUCs between metastatic and non-metastatic groups appears to stabilize, indicating a finite difference at the limit of large sample sizes. Beyond evaluating classification performance through AUCs, it is possible to measure the overall disease-specific pattern captured by the IMF. This can be achieved by computing the mean effect size, which corresponds to the differences in absorbance between the two groups across all wavenumbers. In Fig. 3e, the representation of the mean effect size per stage is presented. A distinct pattern emerges, indicating a consistent and notable increase in the differential fingerprint magnitude with disease progression.

### Infrared fingerprinting and lung cancer patient survival

**Fig. 4:**
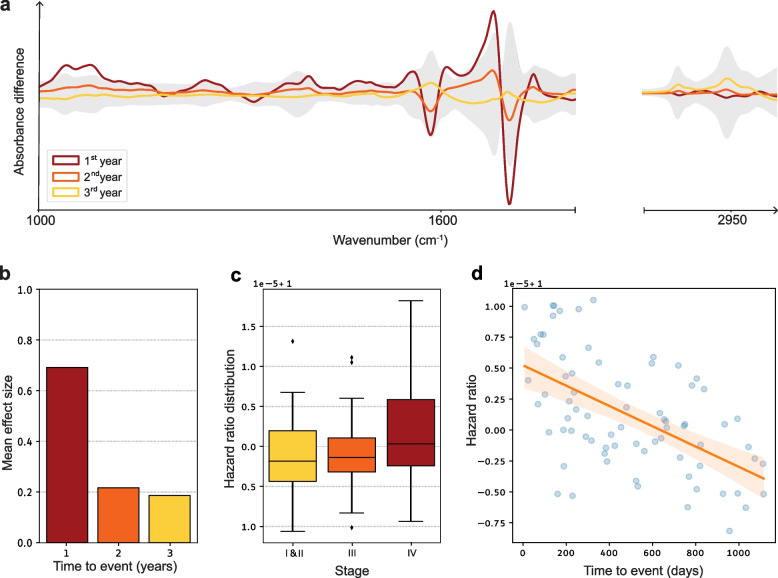
Assessment of information content of IMF concerning patient survival. **a** Differential infrared spectra, showing the mean difference per wavenumber between measured data of non-censored lung cancer patients whose death is observed within 1, 2, or 3 years after the first diagnosis. The control group, in this case, corresponds to censored patients. The shaded area corresponds to the standard deviation per wavenumber in the control group. **b** Mean effect size indicating the overall average differences between fingerprints of non-censored and censored lung cancer patients. The non-censored patients are stratified as in panel (**a**). Information on the cohort characteristics used in the analysis presented in panels (**a**) and (**b**) is provided in Additional file 1: Table S5. **c** Box plots depicting the distributions of hazard rates, predicted by the IMF-based Cox regression models, stratified by different tumor stages. This analysis is based on 95 lung cancer patients with available tumor stage information at the time of diagnosis, as described in Table 1.. **d** Scatter plot showing the value of the hazard rates for all 76 non-censored patients included in the cohort against time to event

Having demonstrated the relation between the amplitude of IMF deviations and lung cancer progression, we proceeded to investigate whether IMF can predict the future survival of lung cancer patients, based on IMF analyses at the time of primary diagnosis. To this end, we fit patient survival regression models using Cox proportional-hazards, based on the IMF data. To assess the prognostic utility of these models and to compare them to models based on other covariates, such as TNM staging, we evaluate their performance using the method of k-fold cross-validation. The performance was measured using Harrell’s C-index - defined as the proportion of observations that the model can order correctly in terms of survival times (see Methods section), with 0.5 indicating random chance and a value of 1.0 corresponding to perfect prediction [44]. Using all 160 patients, a C-index of 0.63 ± 0.12 is achieved, indicating that survival information is indeed encoded in the IMFs. Although tempting to speculate that some spectral positions would be more informative than others, we find that the overall outcome of regression capacity is not based on specific infrared spectral features, but rather utilizes information from the entire spectral range. To get a more general evaluation of the informational content and capacity of IMFs from liquid biopsies, we directly compared the capacity of other diagnostic approaches with IMFs - directly, within the same individuals - for their capacity to capture lung cancer. Table 2 shows the results of the performance comparison between IMF and medically established lung cancer markers, as well as histopathological tissue biopsy (current diagnostic benchmark) for predicting the survival of lung cancer patients. The comparison is done by evaluating Harrell’s C-index on trained Cox regression models within a 10-fold cross-validation. We find that the capacity of IMFs to predict the survival of lung cancer patients at primary diagnosis is on par with the capacity of histopathological examination (stage) that requires invasive tissue sampling. Moreover, it is very encouraging to reveal that the IMFs’ capacity is on par with other biomarkers implemented in clinical diagnostics (e.g., NSE (neuron-specific enolase), CEA (carcinoembryonic antigen) CYFRA-21-1 (cytokeratin-fragment-21-1)), revealing the validity and the potential of the evaluated approach. We further analyze the survival information encoded in the IMFs. Here we investigate how spectra of non-censored individuals, who passed away within either 1, 2, or 3 years from primary diagnosis, differ from the censored patients. In Fig. 4a, the graphical representation illustrates the differential infrared spectra. This depiction highlights the average variance per wavenumber among the recorded data of lung cancer patients without censoring, whose demise is observed within 1, 2, or 3 years post the initial diagnosis. The control group in this context comprises censored patients. The shaded region encapsulates the standard deviation per wavenumber within the control group. These results newly identify that the closer a patient is to eventual death, the more pronounced the lung cancer-specific pattern detected via IMF. The same conclusion can be drawn by calculating the effect size (averaged across all wavenumbers) as shown in Fig. 4b. Information on the patient groups included in this analysis is given in Additional file 1: Table S5.

**Table 2:** Performance comparison between infrared molecular fingerprint (IMF) information, and diagnostically established lung-cancer markers for predicting lung cancer patients’ survival performed on the same patients’ blood samples

Parameter	C-index Markers	C-index IMFs	# Patients
IMFs		0.63 $\pm$ 0.12	160
Stage	0.67 $\pm$ 0.16	0.68 $\pm$ 0.12	95
NSE	0.64 $\pm$ 0.15	0.65 $\pm$ 0.12	132
CEA	0.59 $\pm$ 0.18	0.66 $\pm$ 0.12	147
CYFRA-21-1	0.72 $\pm$ 0.10	0.68 $\pm$ 0.10	144
Hemoglobin	0.57 $\pm$ 0.10	0.64 $\pm$ 0.09	159
Leukocytes	0.63 $\pm$ 0.12	0.64 $\pm$ 0.09	159

The number of patients varies across evaluations since not all blood-based parameters were obtained for all of the 160 patients that were included in the cohort study. Therefore, the IMF evaluation for the largest population is given first (*n:160*), with further comparisons of IMF’s performance with different established analytes (on smaller sample sets). The comparison is performed by evaluating Harrell’s C-index on trained Cox regression models within a 10-fold cross-validation

To further investigate the capacity of the hazard prediction by the IMF-based Cox regression models, in terms of the initial stage of patient diagnosis, we used predicted hazard rates as a relative gauge of mortality risk. In Fig. 4c, we illustrate the association between the distribution of hazard rates based on IMFs and the tumor TNM stage. Our findings reveal a noticeable trend (still not statistically significant), with the risk of mortality, as indicated by IMF analysis, exhibiting an increase with higher stages. This trend is better quantified by correlation analysis. By direct calculation one obtains a Point-Biserial correlation between hazard ratios and stage, which in this case is $\rho = 0.31$ with a *p*-value $p=0.02$. Even if not a fully significant result, the observed correlation reaffirms the above-established association between IMFs and disease progression. In addition, we explore the relation between the predicted hazard rates and the time to observed death, for non-censored patients. Figure 4d shows a scatter plot of the value of the hazard rates, for all 76 non-censored patients included in the cohort, against time to event. We observe a clear correlation between the hazard rates and the time to event. This is also confirmed by direct calculation of the Pearson correlation, which in this case is $\rho = 0.56$ with a *p*-value $p = 1.5 \times 10^{-7}$.

## Discussion

The potential of liquid biopsies to contribute to survival outcome prediction and to possibly aid in stratifying lung cancer patients has been anticipated [45]. However, the technical limitations for the clinical utility of liquid biopsies for early-stage NSCLC have still not been solved [46]. Infrared spectroscopy - which provides multi-molecular information in a cost- and time-effective manner - carries great potential for minimally invasive analysis, and has not been evaluated for this application before. We previously revealed that infrared molecular fingerprinting has a fair capacity to detect four common cancers based on spectroscopy of venous blood plasma and serum [12]. In the present study, we extend the paradigm and evaluate whether infrared fingerprinting of liquid biopsies has any power to predict possible patient outcomes at the time of primary diagnoses, specifically focusing on survival. We analyze 160 treatment-naive lung cancer patients, follow them over time, and quantify the survival rates of 84 censored and 76 non-censored individuals. This is the first example that IMF has been robustly evaluated for survival prognostication of any cancerous lesion. Interestingly, we find that our approach is statistically *on par* with histopathological stage information and established diagnostic molecular markers to predict the survival of lung cancer patients. Furthermore, we showed that IMF could be conceptually considered as a set of markers that when combined are associated with patient outcomes, such as disease progression, and survival.

A limitation of the present study is that the approach has only been evaluated on a single, albeit fairly sizable cohort. To address this, we are planning an independent validation study to test whether this in vitro profiling assay would work well on individuals with diverse genetic and lifestyle factors. Additionally, therapeutic efficiency and effects related to single or combined lung cancer therapies that patients receive have certainly contributed to the observed survival rates. In the present comparisons, we have pooled individuals receiving various medications and did not evaluate their impact, which is beyond the scope of the current study. Nevertheless, all patients received the best available therapy, making them sufficiently comparable for the analyses performed in this study.

Another area where IMFs could be instructive is the stratification of individuals at a higher risk of developing lung cancer [47]. Plasma-based omics have already been shown to provide a means to predict individuals at higher risk of future lung cancer [48, 49]. Our results could make a significant contribution in this avenue if evaluated and tested in longitudinal settings. One potential advantage is that the IMFs could provide insight both into the individual’s risk of lung cancer and a means of detecting imminent early-stage disease. IMF may have an advantage here - as other tests that rely on biomarkers released by cancer cells into blood face the challenge of low abundance in early-stage disease. Furthermore, they are also less likely to reflect inherent cancer risk in the same way as biomarkers that capture host factors by our “molecularly holistic” approach covering - e.g., smoking-induced damage or immune response to precursor lesions and early tumors.

## Conclusions

This study highlights the potential of infrared molecular fingerprinting as a novel, minimally invasive tool for predicting survival outcomes and assessing disease progression in treatment-naive lung cancer patients. By evaluating IMFs from a well-characterized cohort, we demonstrated that this approach offers predictive capabilities comparable to histopathological staging and established molecular markers. Importantly, this is the first investigation to robustly link IMF profiles to survival outcomes in cancer, showcasing its promise as a diagnostic and prognostic aid.

Our findings underscore the utility of IMFs in capturing the complex molecular landscape of lung cancer, which may reflect not only tumor-specific factors but also host responses and disease-related physiological changes. This holistic profiling approach holds particular promise for addressing current limitations in liquid biopsy technologies, especially in the context of early-stage disease and survival prognostication.

Nevertheless, further validation studies are needed to confirm the generalizability of these results across diverse populations and clinical settings. Future research should also explore the integration of IMF-based analyses with therapeutic monitoring and risk stratification tools to enhance their clinical applicability. By advancing this field, IMF technology could contribute to improving early detection, personalized treatment planning, and ultimately, patient outcomes in lung cancer.

## Supplementary Information

Additional file 1. Additional file 1: Tables S1-S5. Table S1 – details on laboratory parameters. Table S2 – numbers of patients with various comorbidities. Table S3 – cohort breakdown in terms of three classesused in the definition of patient stages, following the TNM Classification of Malignant Tumors. Table S4 – *p*-values for comparing survival functions related to different TNM tumor stages using log-rank testing. Table S5 – characteristics of the lung cancer cohort used in the analysis related to the results shown in Fig. 4a and b.

## Data Availability

No datasets were generated or analysed during the current study.

## Abbreviations

FTIR: Fourier-transform infrared
LDCT: Low-dose computed tomography
IR: Infrared
IMF: Infrared molecular fingerprint
TNM: Tumor, node, metastasis
LMU: Ludwig-Maximillian-University
ICH-GCP: Good clinical practice
DRKS: German clinical trials register
CPC: Comprehensive pneumology centre
NSCLC: Non-small-cell lung cancer
BMI: Body-mass-index
UICC: Union for International Cancer Control
SCC: Squamous cell carcinoma
AC: Adenocarcinoma
LCC: Large-cell carcinoma
CIs: Confidence intervals
ROC: Receiver operating characteristic
AUC: Area under the curve
GLM: Generalized linear models
NSE: Neuron-specific enolase
CEA: Carcinoembryonic antigen
CYFRA: Cytokeratin fragment
