# Correction: Assessing lung cancer progression and survival with infrared spectroscopy of blood serum

**DOI:** 10.1186/s12916-025-03991-6

**Published:** 2025-03-23

**Authors:** Kosmas V. Kepesidis, Mircea-Gabriel Stoleriu, Nico Feiler, Lea Gigou, Frank Fleischmann, Jacqueline Aschauer, Sabine Eiselen, Ina Koch, Niels Reinmuth, Amanda Tufman, Jürgen Behr, Mihaela Žigman

**Affiliations:** 1 Chair of Experimental Physics - Laser Physics, Ludwig-Maximilians-Universität München (LMU), Garching, Germany; 2 Laboratory for Attosecond Physics, Max Planck Institute of Quantum Optics (MPQ), Garching, Germany; 3 Center for Molecular Fingerprinting (CMF), Budapest, Hungary; 4 Asklepios Biobank for Lung Diseases, Department of Thoracic Surgery, Member of the German Center for Lung Research, DZL, Asklepios Fachkliniken München-Gauting, Munich, Germany; 5 Department of Medicine V, LMU University Hospital, LMU Munich, Member of the German Center for Lung Research, Munich, Germany

**Correction** **:** **BMC Med 23, 101 (2025)**

**https://doi.org/10.1186/s12916-025-03924-3**

The production team that handled this manuscript mistakenly omitted Mihaela Žigman as a co-Corresponding Author. Dr. Žigman has since been restored as a co-Corresponding Author.

Additionally, a formatting error was introduced by the production team in Table 1 during the manuscript’s preparation for publication. This formatting error has now been corrected [1].
