# Supplementary material for: Assessing lung cancer progression and survival with infrared spectroscopy of blood serum
Source: BMC Med. 2025 Feb 21;23:101. doi: 10.1186/s12916-025-03924-3 (PMC11846347; doi:10.1186/s12916-025-03924-3)
Supplement: Supplementary file 1 — Additional file 1. Additional file 1: Tables S1-S5. Table S1 – details on laboratory parameters. Table S2 – numbers of patients with various comorbidities. Table S3 – cohort breakdown in terms of three classesused in the definition of patient stages, following the TNM Classification of Malignant Tumors. Table S4 – p-values for comparing survival functions related to different TNM tumor stages using log-rank testing. Table S5 – characteristics of the lung cancer cohort used in the analysis related to the results shown in Fig. 4a and b. [file 12916_2025_3924_MOESM1_ESM.pdf]

# Supplementary Tables for “Assessing lung cancer progression and survival with infrared spectroscopy of blood serum”

Additional file 1: Tables S1–S5. These additional tables contain further details on the cohorts of study participants described in Table 1. Table S1 gives details on laboratory parameters. Table S2 lists the number of patients with various comorbidities. Table S3 shows the breakdown of the cohort in terms of three classes (T - tumor size, N - regional lymph node involvement, M - metastasis) used in the definition of patient stages following the TNM Classification of Malignant Tumors. Table S4 displays the p-values for comparing survival functions related to different TNM tumor stages using log-rank testing. Finally, table S5 shows the characteristics of the lung cancer cohort used in the analysis related to the results shown in Figure 4 (a) and (b) in the main text.

| | Censored | Non-censored |
|---|---|---|
| Leukocytes (#/nL) | $8.2 \pm 2.18$ | $9.65 \pm 3.18$ |
| Thrombocytes (#/nL) | $277.13 \pm 72.76$ | $317.08 \pm 95.48$ |
| Hemoglobin (g/dL) | $13.99 \pm 1.71$ | $13.73 \pm 1.42$ |
| Creatinine (mg/dL) | $0.92 \pm 0.29$ | $0.94 \pm 0.28$ |
| GFR (mL/min) | $77.43 \pm 17.55$ | $76.34 \pm 19.29$ |
| GPT (U/L) | $27.02 \pm 9.91$ | $27.08 \pm 14.51$ |
| GOT (U/L) | $25.3 \pm 23.36$ | $24.16 \pm 15.93$ |
| LDH (U/L) | $211.22 \pm 75.89$ | $235.93 \pm 96.55$ |
| Cholesterol (mg/dL) | $195.24 \pm 35.99$ | $197.07 \pm 43.45$ |
| Albumin (g/dL) | $3.76 \pm 0.36$ | $3.52 \pm 0.49$ |
| Cyfra 21-1 (ng/mL) | $4.69 \pm 11.29$ | $8.19 \pm 12.99$ |
| NSE (ng/mL) | $18.03 \pm 5.08$ | $23.35 \pm 15.19$ |

**Table S1:** Distributions of laboratory parameters between censored and non-censored lung-cancer patients, included in the lung-cancer cohort, studied in the survival analysis.

| | | Censored | Non-censored |
|---|---|---|---|
| Diabetes | Yes | 17 | 16 |
| | No | 66 | 60 |
| | N/A | 1 | 0 |
| High blood pressure | Yes | 45 | 45 |
| | No | 36 | 31 |
| | N/A | 3 | 0 |
| Heart disease | Yes | 22 | 25 |
| | No | 59 | 50 |
| | N/A | 3 | 1 |
| Kidney disease | Yes | 5 | 5 |
| | No | 79 | 71 |
| | N/A | 0 | 0 |
| Rheumatic disease | Yes | 2 | 5 |
| | No | 81 | 70 |
| | N/A | 1 | 1 |
| Fibrosis | Yes | 0 | 0 |
| | No | 26 | 36 |
| | N/A | 58 | 38 |
| Asthma | Yes | 2 | 5 |
| | No | 25 | 33 |
| | N/A | 57 | 38 |
| COPD | Yes | 25 | 31 |
| | No | 2 | 7 |
| | N/A | 57 | 38 |
| COPD grade | GOLD 1 | 2 | 8 |
| | GOLD 2 | 13 | 14 |
| | GOLD 3 | 8 | 5 |
| | GOLD 4 | 1 | 2 |
| | N/A | 60 | 47 |

**Table S2:** Breakdown of the lung-cancer cohort, studied in the survival analysis, in terms of comorbidities.

| | | Censored | Non-censored |
|---|---|---|---|
| Class T | T1 | 30 | 15 |
| | T2 | 27 | 21 |
| | T3 | 13 | 10 |
| | T4 | 13 | 25 |
| | N/A | 1 | 5 |
| Class N | N0 | 57 | 26 |
| | N1 | 11 | 6 |
| | N2 | 6 | 21 |
| | N3 | 9 | 17 |
| | N/A | 1 | 6 |
| Class M | M0 | 33 | 31 |
| | M1 | 9 | 28 |
| | N/A | 42 | 17 |

**Table S3:** Breakdown of the lung-cancer cohort, studied in the survival analysis, in terms of TNM classes.

| Compared stages: | I / II | I / III | I / IV | II / III | II / IV | III / IV |
|---|---|---|---|---|---|---|
| p-value: | 0.53 | 0.11 | ¡0.005 | 0.09 | 0.01 | 0.05 |

**Table S4:** P-values for comparing survival functions related to different TNM tumor stages using log-rank testing

| years to event | # patients | Age (years) | % Female |
|---|---|---|---|
| 1 | 33 | $69 \pm 10$ | 33 |
| 2 | 23 | $71 \pm 11$ | 48 |
| 3 | 18 | $68 \pm 12$ | 50 |
| censored | 84 | $68 \pm 9$ | 46 |

| years to event | Stage I | Stage II | Stage III | Stage IV | N/A |
|---|---|---|---|---|---|
| 1 | 1 | 2 | 8 | 18 | 4 |
| 2 | 2 | 0 | 6 | 7 | 8 |
| 3 | 2 | 0 | 4 | 3 | 9 |
| censored | 11 | 7 | 13 | 9 | 44 |

**Table S5:** Characteristics of the lung cancer cohort used in the analysis related to the results presented in Figure 4 (a) and (b). The first part shows the breakdown of lung cancer patients in terms of demographics. The second part shows tumor staging information according to the TNM Classification of Malignant Tumors (Union for International Cancer Control (UICC)).
